# The neurocognitive correlates of academic diligence in adolescent girls

**DOI:** 10.1080/17588928.2018.1504762

**Published:** 2018-08-27

**Authors:** Delia Fuhrmann, Susanne Schweizer, Jovita Leung, Cait Griffin, Sarah-Jayne Blakemore

**Affiliations:** a Institute of Cognitive Neuroscience, Division of Psychology and Language Sciences, University College London, London, UK; b MRC Cognition and Brain Sciences Unit, School of Clinical Medicine, University of Cambridge, Cambridge, UK

**Keywords:** Academic diligence, adolescence, inferior frontal gyrus, dual systems hypothesis, striatum

## Abstract

Academic diligence is the ability to regulate behavior in the service of goals, and a predictor of educational attainment. Here we combined behavioral, structural MRI, functional MRI and connectivity data to investigate the neurocognitive correlates of diligence. We assessed whether individual differences in diligence are related to the interplay between frontal control and striatal reward systems, as predicted by the dual-systems hypothesis of adolescent development. We obtained behavioral measures of diligence from 40 adolescent girls (aged 14-15 years) using the Academic Diligence Task. We collected structural imaging data for each participant, as well as functional imaging data during an emotional go-no-go self-control task. As predicted by the dual-systems hypothesis, we found that inferior frontal activation and gyrification correlated with academic diligence. However, neither striatal activation nor structure, nor fronto-striatal connectivity, showed clear associations with diligence. Instead, we found prominent activation of temporal areas during the go-no-go task. This suggests that academic diligence is associated with an extended network of brain regions.

Academic diligence is the ability to regulate behavior in the service of academic goals (Galla et al., 2014). Diligence is strongly correlated with personality traits like conscientiousness and grit, all of which have been shown to predict educational attainment (Credé, Tynan, & Harms, 2016; Duckworth & Gross, 2014; Duckworth, Peterson, Matthews, & Kelly, 2007; Galla et al., 2014). The contribution of diligence to educational attainment is thought to be dissociable from, and sometimes orthogonal to, measures such as fluid ability or intelligence (Duckworth et al., 2007).

It has been proposed that, at a mechanistic level, diligence is the product of two conflicting psychological processes—the exercise of will and the drive to seek immediate gratification (Duckworth & Steinberg, 2015). Adolescence may be a time during which this conflict is particularly acute (Casey, Getz, & Galván, 2008; Steinberg, 2008; Steinberg et al., 2017): The ability to regulate one’s behavior is thought to develop gradually between childhood and adulthood and mature in the mid-twenties (Steinberg et al., 2017). In contrast, the drive to seek novel and rewarding experiences is thought to develop non-linearly and peak in adolescence (Steinberg et al., 2017). This ‘imbalance’ between self-control and reward-sensitivity during adolescence has been proposed to originate in the relatively early maturation of the subcortical reward system combined with a slower and more protracted development of frontal control systems (Casey et al., 2008; Steinberg, 2008).

This dual-systems hypothesis of frontal self-control and striatal reward systems has been one of the most influential models of adolescent development (Shulman et al., 2016) and has been used to explain phenomena such as increased risk-taking and sensation-seeking in adolescence (Casey et al., 2008; Steinberg, 2008; Van Den Bos, Rodriguez, Schweitzer, & McClure, 2015). There has been some empirical support for this theory. Somerville and colleagues (2011) used an emotional go-no-go task as a measure of self-control and showed that inferior frontal activation and connectivity correlated with response inhibition in children, adolescents and adults. Adolescents, compared with children and adults, additionally showed increased activation of  the ventral striatum, which was linked to non-linear reductions in impulse control to rewarding cues (happy faces), while response inhibition to neutral cues (neutral faces) improved linearly with age. This development of inhibitory control was taken as evidence that adolescents find it harder than other age groups to resist responding to rewarding social cues (Somerville, Hare, & Casey, 2011).

While many studies find similarly increased average impulsivity and reduced self-control in adolescence, compared with other ages (Braams, Van Duijvenvoorde, Peper, & Crone, 2015; Casey et al., 2008; Steinberg, 2008), the dual-systems hypothesis in its original form has been critiqued as overly simplistic (Casey, Galván, & Somerville, 2016; Crone & Dahl, 2012; Pfeifer & Allen, 2012): Imaging studies have highlighted heterogeneity in the development of frontal and striatal structures (Pfeifer & Allen, 2012), task-related functional activation (Crone & Dahl, 2012) and individual trajectories of structural maturation (Foulkes & Blakemore, 2018; Mills, Goddings, Clasen, Giedd, & Blakemore, 2014). Such individual differences are relevant to education and may predict inter-individual variance in academic diligence (Duckworth & Steinberg, 2015). Therefore, individual differences in diligence might be hypothesized to correlate with fronto-striatal structure and function.

Here, we investigated the neurocognitive correlates of diligence in adolescent girls. We assessed whether individual levels of diligence are related to fronto-striatal structure, function and connectivity in 40 girls aged 14–15 years. We chose this age group because previous studies have highlighted that mid-adolescents may find self-control tasks particularly challenging (Braams et al., 2015). A relatively narrow age range was chosen so as to not confound individual differences in self-control with the on-going development of executive functions during adolescence (Baum et al., 2017). We chose to recruit only girls because of sex differences in pubertal development (Sisk & Foster, 2004).

We obtained behavioral measures of diligence using the Academic Diligence Task (ADT), which is designed to model participants’ behavior when doing school-work (Galla et al., 2014). In this task, participants can freely allocate their time between doing boring math exercises (which participants are told are beneficial for learning) and playing entertaining video games. The task has been shown to have incremental predictive validity for educational outcomes such as Grade Point Averages and performance on standardized math and reading tests (Galla et al., 2014). Questionnaire measures of related constructs such as grit and self-control also reliably predicted unique variance in task behavior, whereas agreeableness, a personality trait encompassing compliance, did not, thus demonstrating convergent and discriminant validity of this task (Galla et al., 2014). We investigated how behavior on the ADT was related to structure, function and connectivity of the inferior frontal gyrus and the striatum. We collected functional imaging data during an emotional go-no-go task with happy and neutral peer faces as cues (Somerville et al., 2011). We chose a go-no-go task as an established measure of self-control, which has been consistently associated with activation in well-defined frontal (inferior frontal gyrus) and striatal (ventral and dorsal) regions of interest (Ahmed, Bittencourt-Hewitt, & Sebastian, 2015; Simmonds, Pekar, & Mostofsky, 2008; Somerville et al., 2011). Using this task allowed us to interpret our findings in relation to previous studies with adults and adolescents and to test whether neural activation in the go-no-go task is associated with behavior on more naturalistic self-control tasks like the ADT. We chose the emotional variant of the go-no-go task as adolescents may be particularly responsive to emotional face stimuli (Somerville et al., 2011) and affective stimuli in general (Prencipe et al., 2011).

Based on the dual-systems hypothesis and previous go-no-go studies (Casey et al., 2008; Somerville et al., 2011; Steinberg, 2008), we predicted that increased functional activation of the inferior frontal gyrus and decreased activation of the ventral striatum in the go-no-go task would correlate positively with diligence. We further predicted that increased diligence would be associated with increased connectivity strength between the inferior frontal gyrus and dorsal striatum, as well as decreased gray matter volume in the inferior frontal gyrus and the striatum.

## Methods

### Participants

Forty-two typically developing girls aged 14–15 years were recruited for this study. Participants attended eight different schools in Greater London and Cambridgeshire and were recruited through advertisements in schools and on social media. Twenty-eight participants attended state schools and 14 participants attended private schools. Participants were tested during a 2-hour testing session that included behavioral and neuroimaging assessments (see below for details). Two participants were excluded from all analyses because of excessive head motion in the scanner (see Imaging data acquisition and pre-processing), leaving a total of 40 participants in the analyzed sample (Table 1).

During behavioral assessment we administered cognitive tests, questionnaire measures and the ADT (Galla et al., 2014; see Behavioral task). IQ was measured using the WASI (Wechsler Abbreviated Scale of Intelligence, Wechsler, 1999) matrix reasoning subscale. WASI matrix reasoning scores can be transformed to t-scores that, in turn, can be converted to IQ estimates as per normative guidelines. Socio-economic status (SES) was measured by asking participants to report parental education level, a robust indicator of SES (Dubow, Boxer, & Huesmann, 2009). SES was calculated by taking the mean of maternal and paternal education levels.

**Table 1.** Participant Characteristics.

**Age** (in years)	*range*	14.10–15.90
	*M*	14.99
	*SE*	0.09
**IQ**	*range*	85.03–122.51
	*M*	105.68
	*SE*	1.30
**SES**	*range*	2–6
	*median*	5
	*IQR*	1.5

*Note*. SES = socio-economic status; IQR = interquartile range. SES scores: 1 = 1 + O levels/CSEs/GCSEs; 2 = 5 + O levels/CSEs/CSEs; 3 = 1 + A levels/AS levels; 4 = 3 + A levels/AS levels; 5 = First Degree (e.g. BA, BSc); 6 = Higher Degree (e.g. MA, PhD)

The study was carried out in accordance with UCL Research Ethics Guidelines and was approved by the UCL Research Ethics Committee. Informed consent from parents and assent from all participants was obtained.

### Behavioral task

**Figure 1.**
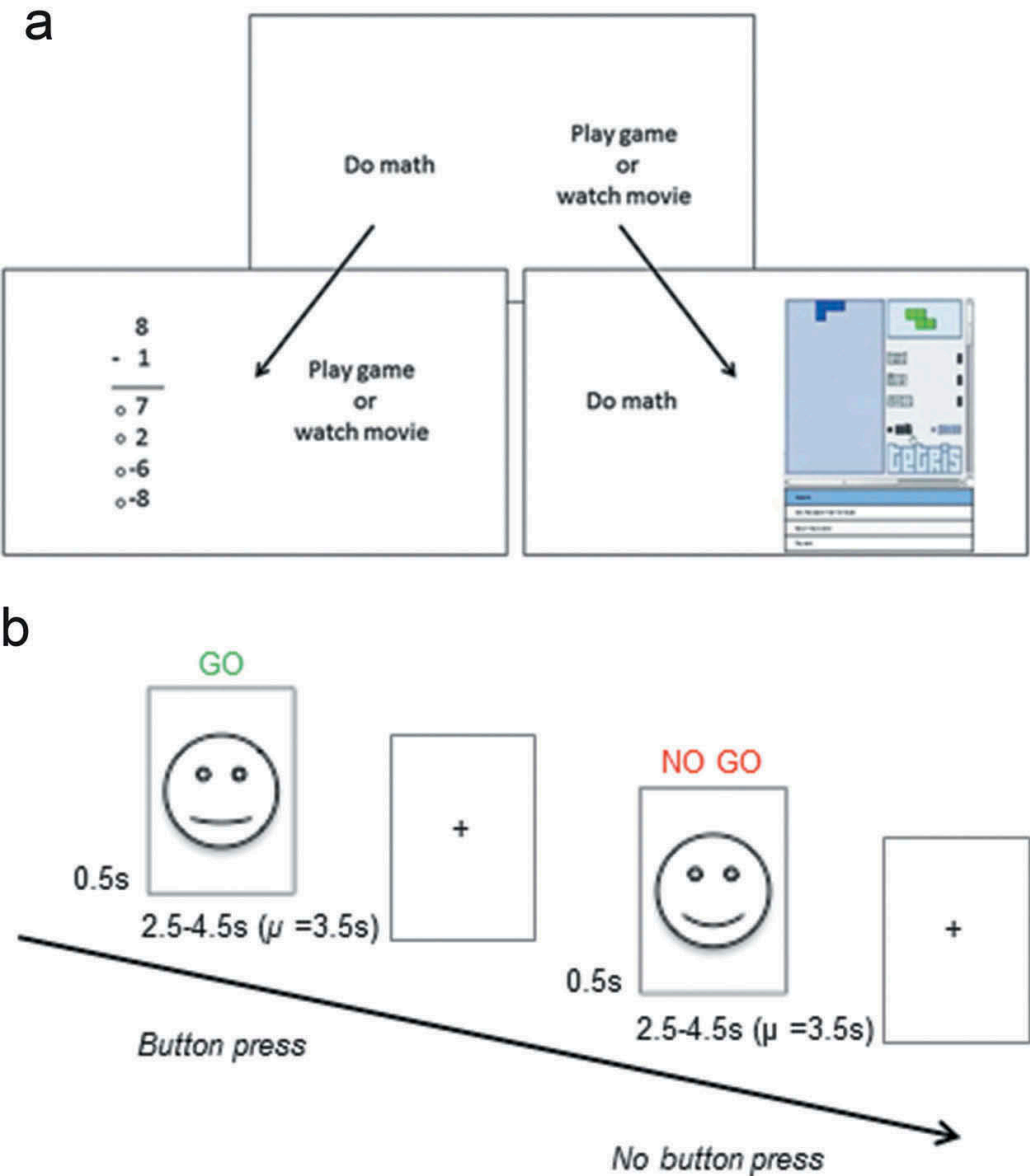
Schematics of the Academic Diligence Task (Panel A; Galla et al., 2014) and the emotional go-no-go task (Panel B; Somerville et al., 2011). Figure 1A was adapted from Galla et al. (2014), © Elsevier, all rights reserved. Reproduced here with permission from Elsevier. Face stimuli in Figure 1B are shown here schematically only. Face stimuli for the actual task were photographs of real faces obtained from the NIMH-ChEFS adolescent face stimulus set (Egger et al., 2011).

The *Academic Diligence Task* (Galla et al., 2014) is designed to mirror real-world choices students face when completing school-work. The task is available for preview and download as freeware (https://angeladuckworth.com/research/academic-diligence-task/and) and was used, unaltered, for the purpose of this study. The ADT consists of a split-screen interface with the choice to play games (Tetris_TM_ etc.) or complete simple single-digit arithmetic problems (Figure 1A; e.g. 6 + 1, 5 × 2 etc.). Arithmetic problems were kept at a simple level for two reasons. First, the task was purposefully designed to be boring so as to adequately capture the conflict between the temptation to engage in an interesting distraction (play games), versus the desire to fulfill a necessary but tedious task (boring math). Using extremely simple and boring math problems reduced the likelihood that students—even those who normally enjoy math—would find these tasks engaging. Second, the task was kept simple so that diligence was not confounded with math ability: Adolescent participants were expected to perform at ceiling in the math problems, ensuring that variation in the time spent on math problems was due to diligence rather than math ability.

Participants were first shown an introduction screen that highlighted the alleged benefits of practicing math equations: ‘New scientific research shows that students who practiced math by doing more subtraction, addition and multiplication problems went on to earn higher grades. Even doing simple and easy math problems can make you a better problem solver, which can help you in all areas of your life.’ Participants then practiced arithmetic problems before being instructed to solve as many math problems as quickly and accurately as possible in the main task. They were also told that they always had the option to take a break and play games: ‘Remember, you will be able to play games whenever you feel like it, but the more problems you do, the better you will become at problem solving’. Participants then completed three blocks during which they could freely allocate their time between math and games. After each block, participants were asked to rate how bored they felt on a 5-point scale (1 = not at all bored to 5 = very bored). After the last block, participants also rated how tempting they found the games on a 5-point scale (1 = not at all temping to 5 = very tempting).

Once instructions for the ADT had been given by an experimenter, participants were left to complete the task on their own in the testing room. This procedure was implemented to reduce demand characteristics: We wanted to minimize the chance of the participant thinking they should complete the math just because the experimenter was present. Participants were also informed before the study that data would be stored anonymously and not shared with schools or anyone else. The experimenter was available immediately outside the testing room in case the participant had any questions, and participants were aware of this. We invited two participants to the lab at a time. One participant completed the behavioral tasks while the other completed the neuroimaging component, after which they switched. The order of behavioral assessments and neuroimaging was thereby counterbalanced between participants. The ADT took 20 minutes in total, and we operationally defined *diligence* as the percentage of time participants spent doing math.

### fMRI task

We used an *emotional go-no-go* task (Somerville et al., 2011) to measure self-control (Figure 1B). Participants were presented with happy or neutral faces and were instructed to respond to one of them by clicking a button (go stimulus, e.g. neutral faces) and not respond to the other (no-go stimulus, e.g. happy faces). We used adolescent faces as stimuli to reflect the importance of peers in this age group (Crone & Dahl, 2012). The stimuli were 18 girls’ faces (happy and neutral expression for each) obtained from the NIMH-ChEFS adolescent face stimulus set (Coffman et al.,).

Two-thirds of the face stimuli were go stimuli and one-third were no-go stimuli. This weighting was used to make the frequent go stimuli the pre-potent response and to increase the difficulty of inhibiting responses on infrequent no-go stimuli (Simmonds et al., 2008). Participants completed go-no-go conditions in which happy faces were the frequent go and neutral faces the infrequent no-go stimuli, and conditions in which neutral faces were the frequent go and happy faces the infrequent no-go stimuli.

Go-no-go blocks were interspersed with never-go blocks during which participants passively viewed faces. These blocks were used to control for potential confounds (see Functional imaging analyses). Stimulus frequency in never-go blocks was weighted just as in go-no-go blocks (i.e. blocks contained either infrequent happy and frequent neutral, or frequent happy and infrequent neutral faces).

Participants completed one functional run in which two-thirds of stimuli were happy faces, and one run in which two-thirds of stimuli were neutral faces. The order of the runs was counterbalanced between participants. Each of these runs consisted of eight blocks, four of which were go-no-go blocks and four never-go blocks. Each block consisted of 12 trials. A fixation cross was presented during a jittered (2000–7000 ms, *M *= 4500 ms) inter-stimulus interval. Each functional run took 8 min in total. The task was presented and responses were acquired with Cogent 2000 (Cogent 2000 Team, 2015) and Matlab (The MathWorks, 2013).

In order to test whether happy stimuli are actually rewarding, after the scanning session we asked participants to rate how much they liked looking at each of the face stimuli on a 7-point scale (1 = like a great deal to 7 = dislike a great deal). For the analysis, ratings were centred at 0 and inverted for ease of interpretation.

### Imaging data acquisition and pre-processing

Imaging data were acquired using Siemens Avanto 1.5T MRI scanner. We ran a structural sequence (T1-weighted, 64 slices, TR = 1.17 s, TE = 0.01 s), two functional runs (T2-weighted, each run: 520 volumes, 44 slices, TR = 1 s, TE = 0.045 s) and a fieldmap in two sequences (each sequence: 64 slices, TR = 1.17 s, TE = 0.01 s). Each participant spent approximately 30 min in the scanner.

Imaging data were pre-processed and analyzed using SPM12 (Wellcome Trust Centre for Neuroimaging, 2014). To allow for T1 equilibration effects, the first eight volumes of each session were discarded. The EPI images were sinc interpolated in time for correction of slice-timing differences. Images were also realigned to the first scan by rigid body transformations to correct for head movements. The field map scans were pre-processed with the FieldMap toolbox (Andersson & Hutton, 2017) and used to correct for magnetic field distortions in functional scan.

EPI images (original voxel size of 3 × 3 × 3 mm^3^) and structural images (original voxel size 1x1x1 mm^3^) were co-registered and normalized to the T1 standard template in Montreal Neurological Institute space. The voxel size specified during normalization was also 3 × 3 × 3 mm^3^ for functional and 1x1x1 mm^3^ for structural scans. Proportional scaling and high-pass temporal filtering with a cut-off of 128 s was applied to remove low-frequency drifts in signal. Images were smoothed using a Gaussian kernel of full-width-half-maximum of 8 mm.

Realignment estimates were used to calculate frame-wise displacement (FD) for each volume, which is a composite, scalar measure of head-motion across the six realignment estimates (Siegel et al., 2014). Volumes with FD > 0.9 mm were censored and excluded from further analysis by including a regressor of no interest for each censored volume in the general linear model (see Functional imaging analyses). Scanning sessions with more than 5% of volumes censored, or a root mean square movement greater than 1.5 mm in any run, were excluded from the analysis. This applied to two participants whose data were excluded from all analyses.

### Functional imaging analyses

Following pre-processing, statistical analyses were conducted on correct trials using a GLM. Activated voxels for inhibition (no-go > go trials) and emotion (happy > neutral trials) were identified using an epoch-related statistical model, convolved with a canonical hemodynamic response function and mean-corrected. The GLM included the main effects of inhibition and emotion, as well as their interaction.

In line with standard fMRI practice (Poldrack, 2007), we first carried out a whole-brain analysis (cluster-level, *p* < .001, false discovery rate (FDR) corrected) to investigate the effects of inhibition, emotion and the interaction between the two. We used an exclusive mask for which we contrasted infrequent never go trials with frequent go trials (*p* = .001). This mask was used to isolate activation due to inhibitory processes and to exclude activation due to the absence of a motor response or to viewing an infrequent stimulus.

To investigate the interaction between diligence, inhibition, and emotion further we extracted activations of *a priori* regions of interests (ROIs) using MarsBaR (Brett, Anton, Valabregue, & Poline, 2002). The ROIs were defined by Somerville et al. (2011) and consisted of 4 mm spheres in the right inferior frontal gyrus (IFG: x = 34, y = 26, z = 0), the dorsal striatum (DS: x = 10, y = 16, z = 4), and the ventral striatum (VS: x = −4, y = 15, z = −13).

### Connectivity analyses

We used psycho-physiological interaction (PPI) analysis to estimate task-related changes in connectivity between the IFG and other brain regions (Wellcome Trust Centre for Neuroimaging, 2014). The PPI analysis involved extracting the BOLD signal from the functional right IFG ROI used in the functional analysis (4 mm sphere, with x = 34, y = 26, z = 0 as peak coordinates) as seed region and forming the interaction term between the source signal and the task conditions. A second GLM analysis was then carried out that included the interaction term between the source signal and the task conditions, the source region’s extracted signal, the experimental factors and the movement regressors as effects of no interest. Participant-specific PPI models were run, and contrast images generated for each condition. These ‘first level’ contrast images were then entered into the full-factorial model to assess connectivity of the IFG during inhibition. We assessed whole brain connectivity (cluster-level, *p* < .001, FDR-corrected) and then extracted connectivity strength for the DS using MarsBaR (Brett et al., 2002). This analysis was chosen *a priori* to correspond with Somerville et al.’s (2011) analysis methods. We additionally explored IFG connectivity with the VS given its functional role in reward processing and structural proximity and cytoarchitectonic similarity with the DS (Haber & Knutson, 2010).

### Structural imaging analysis

We analyzed gray matter volumes within each of our ROIs using the CAT12 toolbox (Dahnke & Gaser, 2016). We estimated total intracranial volume (TIV) using a function provided by Ridgway (Ridgway, 2007). TIV was then added as a covariate into the analysis to correct for differences in head size as recommended by Peelle and colleagues (Peelle, Cusack, & Henson, 2012). Gray matter volume in the IFG, VS and DS ROIs were extracted using MarsBaR (Brett et al., 2002).

### Surface-based morphometry

In additional exploratory analyses, we investigated links between diligence and cortical surface measures of the right IFG (pars triangularis). We chose the pars triangularis because our functional IFG ROI partially overlapped with this anatomical structure and because it has been associated with inhibitory motor control (Liakakis, Nickel, & Seitz, 2011) required in the go-no-go task. We analyzed cortical thickness, surface area (gyrification), and surface complexity (fractional dimension; Yotter, Nenaduc, Ziegler, Thompson & Gaser, 2011) of the right IFG. All of these measures have been shown to provide complimentary information about individual differences and development (Madan & Kensinger, 2016). Cortical thickness is an established surface-based measure known to be associated with conscientiousness (Riccelli, Toschi, Nigro, Terracciano, & Passamonti, 2017), just like gyrification (Riccelli et al., 2017), but gyrification may be particularly sensitive to developmental differences (Klein et al., 2014). Fractal dimension is relatively unexplored in the context of individual differences in self-control. Like gyrification, it indexes surface area, but may yield more precise estimations (Yotter et al., 2011).

We segmented structural images using CAT12 default options. CAT12 uses projection-based thickness to estimate cortical thickness and to create the central cortical surface for the hemispheres. We then extracted a gyrification index and surface complexity index from surface maps (fractal dimension; Yotter et al., 2011). We smoothed the data for visual quality checks using a 15mm kernel for thickness data and 20mm kernel for folding data (surface complexity and gyrification). We then extracted ROI data for the right IFG (pars triangularis) using the Automated Anatomical Labelling Atlas 2009 (Destrieux, Fischl, Dale, & Halgren, 2010).

### Regression models

To investigate the link between diligence and task-dependent BOLD signal, we implemented LMMs analyzing structure, function and connectivity of the IFG, VS and DS using the lme4 package (Bates, Maechler, & Bolker, 2013) in R (R Core Team, 2015). Significance tests were obtained using an omnibus Type III Wald χ^2^ test.

We built separate models for structure, function and connectivity as dependent variables because the structural analyses necessarily contained fewer fixed effects than the other two analyses (i.e. no contrasts for inhibition and emotion). For each ROI, we built one model analyzing functional activation and one model analyzing grey matter volume. For connectivity, we built one model analyzing connectivity between the IFG and DS, and an exploratory model analyzing connectivity between the IFG and VS. Additionally, we explored surface measures of the right IFG (cortical thickness, surface complexity and gyrification).

The functional and connectivity models contained inhibition (no-go vs. go) andemotion (happy vs. neutral) as independent variables and orthogonal, Helmert-coded fixed effects. Diligence was analyzed as an independent variable and coded as a *z*-scored fixed effect in all models. We further included all possible interactions of the fixed effects. Participant ID and school were included as nested random intercepts. These random intercepts were used to reflect the repeated-measures design and the clustered nature of participants tested. Random slopes were not included in any model because their inclusion led to overfitting and non-convergence of models. The models analyzing grey matter volumes and surface measures included diligence as a *z*-scored fixed effect and school only as a random effect. Participant was not included as a random effect here because the structural models contained no repeated measures.

## Results

### Behavior in the academic diligence task

Diligence scores, reflecting percentage of time spent doing math, were high overall (*M* = 84.14%, *SE* = 2.70%) but individual scores ranged from 34.44% to 96.67%. Participants performed well on the simple arithmetic tasks (percentage accuracy: M = 97.86%; SE = 0.26%%, Min = 92.93%). Productivity (as measured by the number of math problems correctly solved), was also high. On average, participants correctly solved 180.75 problems (SE = 8.64), with a minimum of 94 correctly solved problems. Participants found math moderately boring (ratings: *M* = 2.71 out of 5, *SE* = 0.17) and games moderately tempting (ratings: *M* = 2.84 out of 5, *SE* = 0.20). Diligence did not correlate significantly with IQ (*r*(38) = −0.03, *p *= .841) or SES (*r*(31) = −0.06, *p *= .741).

### Behavior in the go-no-go task

Participants rated happy faces in the emotional go-no-go task positively, indicating that they found them rewarding to look at (*M* = 0.58, *SE* = 0.10). Neutral faces, in contrast, received negative ratings, suggesting they were rated relatively less rewarding (*M* = −0.22, *SE* = 0.12). The difference between the two ratings was significant (*t*(37) = 6.76, *p* < .001).

False alarm rates in the go-no-go task were low and did not significantly differ between happy faces (*M *= 6.72%, *SE *= 1.29%) and neutral faces (*M *= 8.13%, *SE *= 1.29%), (*χ^2^*(1) = 0.96, *p = *.328). There was, however, a difference in participants’ reaction times (*t*(39) = −3.38, *p* = .002). Reaction times to happy go trials (*M *= 582.83 ms, *SE *= 22.71 ms) were significantly faster than to neutral go trials (*M *= 622.21 ms, *SE *= 28.16 ms), indicating that participants respond faster to rewarding social cues than to neutral ones.

In an exploratory analysis, we checked whether reaction times on happy go-no-go trials were associated with diligence. Although the direction of the association was negative, as would be expected, the correlation was small and non-significant (*r*(38) = −0.14, *p *= .406).

### Functional activation

**Figure 2.**
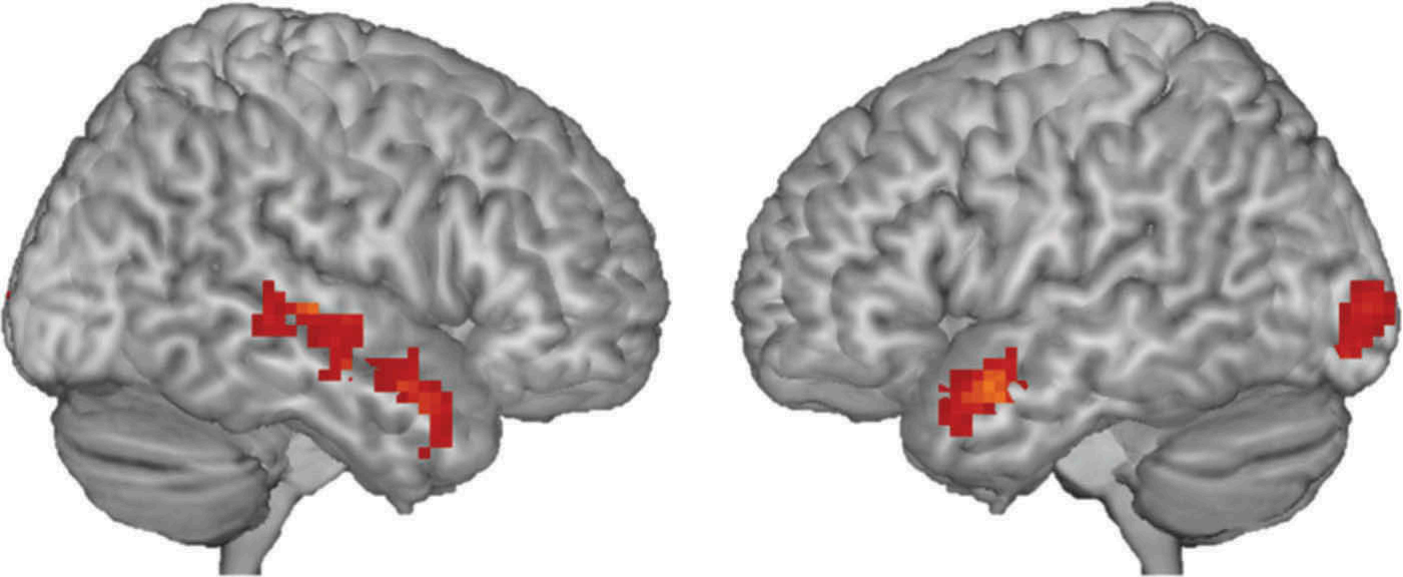
Whole-brain functional activation for the inhibition contrast (no-go > go trials). Results shown are cluster-level FDR-corrected. See Table 2 for significant clusters.

Whole-brain results showed activation of mainly bilateral temporal clusters during inhibition (no-go > go; Table 2, Figure 2). No clusters survived cluster-level FDR-correction for emotion (happy > neutral) or the interaction between inhibition and emotion (see Supplementary Figures 1 to 3 for uncorrected maps).

**Table 2.** Results of the Whole-Brain Analysis of the Inhibition Contrast.

peak activation							
*x*	*y*	*z*	*z*	cluster size	cluster level *p*_FDR_	cluster location	
−51	5	−19	4.64	111	.023	L	middle temporal gyrus and pole, posterior orbitofrontal cortex
51	−22	2	4.47	97	.023	R	middle temporal gyrus
51	8	−25	4.35	87	.023	R	inferior, middle temporal gyrus, middle temporal pole
−27	−97	2	4.17	125	.006	L	inferior occipital gyrus

*Note*. The inhibition contrast is subtracting go from no-go trials. Results shown are cluster-level FDR-corrected.

ROI analyses showed that there were no main effects of inhibition, or emotion for the IFG, VS or DS. There was an interaction between inhibition and emotion for the VS but not the IFG or DS (Table 3). VS activation was lower for happy no-go trials than for happy go trials, while the reverse was the case for neutral trials (Supplementary Figure 4). This indicates that the VS was deactivated more during trials in which participants withheld responded to rewarding cues relative to trials in which they responded to rewarding cues.

**Figure 3.**
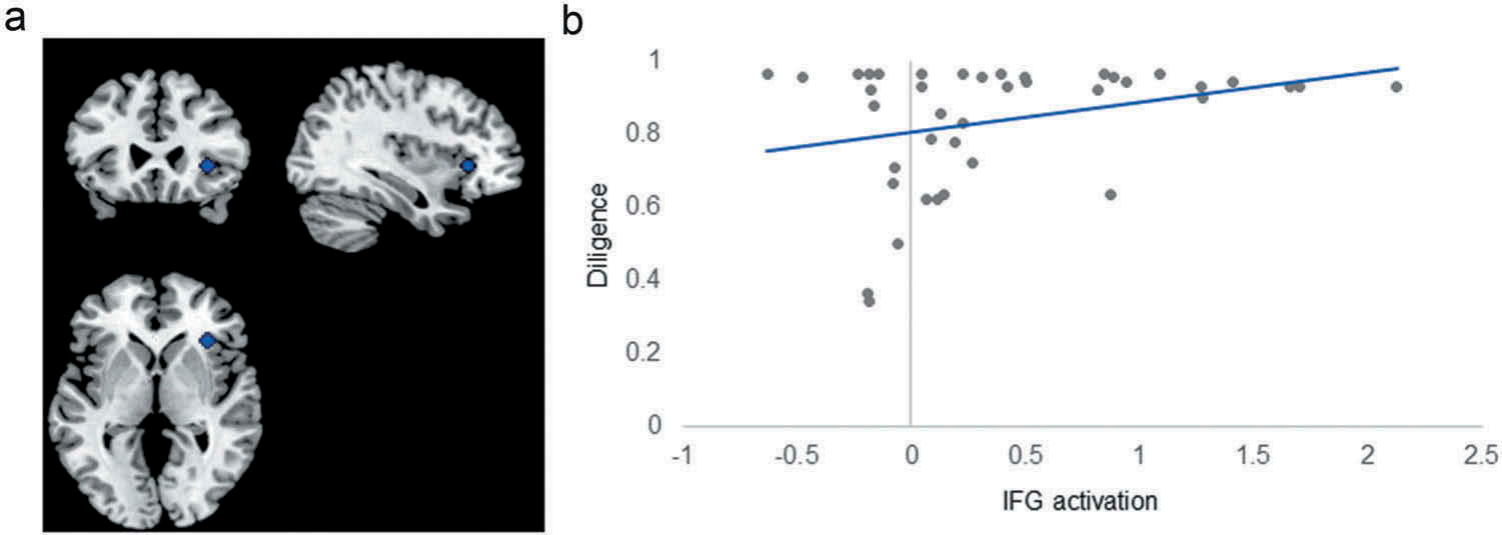
Inferior Frontal Gyrus ROI and Correlation with Diligence. Panel (A) shows the ROI of the inferior frontal gyrus (IFG). Panel (B) shows diligence (proportion of time spent doing math rather than playing games) by average task-dependent IFG activation during the emotional go-no-go. Note that this activation was not specific to task conditions.

Diligence correlated significantly with IFG activation in the task overall, but not with activation of the DS or VS (Table 3). Participants with higher diligence showed higher activation of the IFG during the go-no-go task (β = 0.20; Figure 3). This effect was not moderated by inhibitory load or emotional valence of the stimuli (Table 3).

### Functional connectivity

Connectivity between the IFG and DS was not significantly associated with inhibition, emotion, diligence, or any of their interactions in the whole-brain analysis (see Supplementary Figure 5 for the uncorrected map) and ROI analyses (Table 4). In an exploratory analysis, we additionally investigated connectivity from the IFG to the VS. Similar to connectivity from the IFG to the DS, we found no evidence that connectivity was associated with inhibition, emotion, diligence, or any of their interactions (Table 4).

**Table 3.** Functional Activation of the IFG, DS and VS.

Effect	χ^2^	df	p	
**IFG**				
inhibition	1.87	1	.172	
emotion	0.17	1	.677	
diligence	4.03	1	.045	*
inhibition: emotion	0.62	1	.432	
inhibition: diligence	0.05	1	.833	
emotion: diligence	0.67	1	.412	
inhibition: emotion: diligence	0.30	1	.581	
**DS**				
inhibition	3.07	1	.080	
emotion	0.66	1	.418	
diligence	3.08	1	.079	
inhibition: emotion	1.57	1	.210	
inhibition: diligence	0.18	1	.670	
emotion: diligence	0.84	1	.360	
inhibition: emotion: diligence	1.21	1	.272	
**VS**				
inhibition	0.32	1	.571	
emotion	0.04	1	.845	
diligence	0.25	1	.617	
inhibition: emotion	4.58	1	.032	*
inhibition: diligence	0.11	1	.745	
emotion: diligence	0.28	1	.594	
inhibition: emotion: diligence	0.55	1	.460	

Note.* p < .05

**Table 4.** Connectivity from the IFG seed region.

Effect	χ^2^	df	p
**DS**			
inhibition	3.12	1	.077
emotion	0.00	1	.965
diligence	0.73	1	.393
inhibition: emotion	0.01	1	.911
inhibition: diligence	0.21	1	.646
emotion: diligence	1.14	1	.286
inhibition: emotion: diligence	3.69	1	.055
**VS**			
inhibition	0.31	1	.575
emotion	1.16	1	.281
diligence	0.03	1	.856
inhibition: emotion	0.00	1	.984
inhibition: diligence	0.50	1	.478
emotion: diligence	0.16	1	.691
inhibition: emotion: diligence	0.47	1	.495

### Brain structure

Gray matter volumes did not significantly correlate with diligence for any of our three ROIs (IFG: *χ^2^*(1) = 0.10, *p* = .754; DS: *χ^2^*(1) = 0.80, *p* = .370; VS: *χ^2^*(1) = 0.30, *p* = .584). In exploratory analyses, we investigated the link between IFG cortical structure and diligence. We found that higher diligence was associated with less gyrification of the right IFG (*χ*^2^(1) = 10.50, *p* = .001, β = −0.62). There was no association between diligence and cortical thickness (*χ*^2^(1) = 0.49, *p* = .485) or fractal dimension (*χ*^2^(1) = 0.40, *p* = .530).

## Discussion

The current study investigated the neurocognitive correlates of academic diligence, a predictor of educational attainment, in adolescent girls. We assessed whether individual differences in diligence are related to the interplay between inferior frontal self-control and striatal reward systems, as predicted by the dual-systems hypothesis (Casey et al., 2008; Duckworth & Steinberg, 2015; Steinberg, 2008). Our results were mostly inconsistent with the dual-systems hypothesis. While there was a link between diligence and inferior frontal activation and gyrification, there was no association between diligence and striatal structure and function, or diligence and fronto-striatal connectivity. Instead, we found widespread activation of temporal areas during the go-no-go task.

The functional ROI analysis provided some evidence that frontal activation and structure were associated with diligence, in line with the dual-systems hypothesis (Duckworth & Steinberg, 2015). Activation of the inferior frontal gyrus during the emotional go-no-go task was positively correlated with diligence, although this association was not dependent on inhibitory load or emotional valence. This finding is consistent with resting-state studies linking prefrontal activation to self-control (Gianotti et al., 2009; Knoch, Gianotti, Baumgartner, & Fehr, 2010; Wang et al., 2016) and evidence from previous go-no-go studies showing a positive correlation between right inferior frontal activation and self-control (Simmonds et al., 2008; Somerville et al., 2011). It also complements lesion and correlational studies in adults showing that the personality trait conscientiousness, which is closely related to other measures of self-control (Credé et al., 2016), is associated with lateral frontal functioning (DeYoung et al., 2010; Forbes et al., 2014).

An exploratory analysis further showed that gyrification of the right inferior frontal gyrus was associated with diligence: higher diligence was associated with less gyrification in the right inferior frontal gyrus. This result is in line with previous studies showing that less surface area is associated with higher executive function (Smolker, Depue, Reineberg, Orr, & Banich, 2015) and greater conscientiousness (Riccelli et al., 2017). This supports the notion that the gyrification index may be a more sensitive indicator of individual differences in cognition and development compared to cortical thickness (Klein et al., 2014). Unlike other studies in adults (e.g. Madan & Kensinger, 2016; Yotter et al., 2011), we did not find that fractal dimension was a particularly sensitive measure, but this index of surface area is, at present, underexplored in developmental populations.

Several predictions made by the dual-systems hypothesis were not supported by our data. There was no clear association between diligence and striatal functional activation or structure, or between diligence and inferior frontal gyrus connectivity. We did find that the ventral striatum showed greater activation for trials in which participants responded to happy adolescent faces as compared to trials in which they withheld responses to these faces, while the pattern of activation was reversed for neutral faces. This indicates that the ventral striatum responded preferentially to trials on which participants were looking out for subjectively rewarding social cues. This is in line with previous findings of heightened ventral striatum activation in response to rewards in adolescence (Braams et al., 2015; Somerville et al., 2011). However, this pattern of striatal activation was not directly related to individual differences in diligence in our study.

An unexpected finding was the prominent activation in the temporal cortex during the inhibition task. The whole-brain analysis showed that the emotional go-no-go recruited mainly temporal regions. While many previous inhibitory control studies have mostly focused on frontal regions (Simmonds et al., 2008; Somerville et al., 2011), there are now several go-no-go studies in adolescents that have also shown prominent temporal activation. MEG (Vara et al., 2014; Vara, Pang, Vidal, Anagnostou, & Taylor, 2014) and fMRI studies (Tamm, Menon, & Reiss, 2002) have found that adolescents recruit temporal regions, particularly the right temporal sulcus, more than adults during go-no-go tasks. This recruitment of temporal regions has been proposed to support frontal functioning during development (Vara et al., 2014). Future studies may benefit from probing the interaction between more extended networks than just fronto-striatal systems to better understand the development of self-control during adolescence.

It is possible that some of our null findings are due to limitations of the sample included or tasks used here. There was a range of individual diligence scores (34.44% to 96.67%), but diligence was high overall in our sample: participants chose to do simple and boring math over playing games 84.14% of the time, on average. We cannot rule out the possibility that this skew is due to social desirability or demand characteristics. On the other hand, as a construct, academic diligence may not be entirely separable from social desirability. Diligence may reflect internalized academic drive, or it may reflect a desire to please others, such as parents and teachers, or a mixture of both. We cannot disentangle these explanations at this stage but it is a fascinating question for future research. It is also possible that a larger sample size with even more variability in diligence is needed to detect a stronger correlation between diligence and brain structure and function. This possibility should be investigated in future studies—so far only a handful of studies have investigated neural correlates of diligence and related constructs and very few have probed striatal functioning (Myers, Wang, Black, Bugescu, & Hoeft, 2016; Nemmi, Nymberg, Helander, & Klingberg, 2016).

Another limitation of this study is the limited amount of information available on the subjective experience of the emotional go-no-go and particularly the ADT. We directly probed how participants perceived our emotional go-no-go stimuli and found evidence that happy, but not neutral, faces were perceived as rewarding. The ADT also included questionnaire items probing participants’ boredom during math and temptation by games: our participants found math relatively boring and games relatively tempting. Similarly, an earlier, larger validation study by Galla and colleagues (2014) used multilevel growth curve models to show that boredom during the ADT increased over time if participants chose math, but not if they chose games. They also found that higher levels of boredom and temptation were linked to lower diligence. The ADT could be improved in future studies by including the same questionnaire items (temptation and boredom) for both math and games to allow for a more direct comparison. It would also be useful to assess aptitude for and attitudes toward math, which has been shown to be associated with time spent on mathematic tasks (Singh, Granville, & Dika, 2002). Nonetheless, the evidence available to date suggests that the task is likely to capture every-day conflicts between the wish to pursue educational goals and the temptation to engage in more pleasurable distractions.

Despite these limitations, our study has some implications for adolescent self-control. It echoes previous theoretical work highlighting the limited ability of the dual-systems framework to explain the wide range of adolescent self-control observed in naturalistic settings (Crone & Dahl, 2012; Pfeifer & Allen, 2012). In conjunction with previous research, it also highlights that it may be useful to move away from the duality of fronto-striatal systems and instead explore more extended brain networks (Baum et al., 2017; Vara et al., 2014). Finally, our study underlines that findings from one imaging measure (in this case, functional activation) may not necessarily generalize to other imaging measures (i.e. connectivity and some measures of brain structure). This highlights the need for future, larger, multivariate studies investigating self-control systems systematically across different measures of brain maturation.

## Supplementary Material

Supplemental Material
