# Supplementary material for: The neurocognitive correlates of academic diligence in adolescent girls
Source: Cogn Neurosci. 2018 Aug 27;10(2):88–99. doi: 10.1080/17588928.2018.1504762 (PMC6373776; doi:10.1080/17588928.2018.1504762)
Supplement: Supplemental Material [file PCNS_A_1504762_SM2320.zip › CN_SupplementaryMaterial_final.pdf]

Suplementary Figures

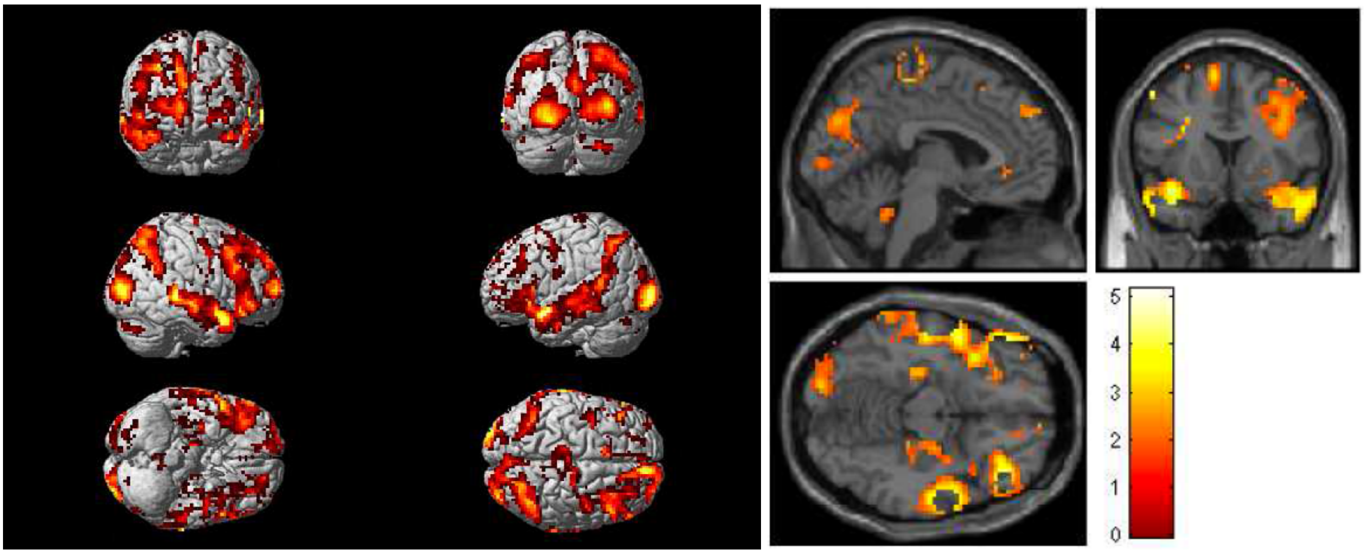

Supplementary Figure 1. Whole-brain functional activation for the inhibition contrast (no-go > go trials). The left panel shows activations in cortical areas, the right panel shows slices at $x = -4$, $y = 15$, $z = -13$ (the center of the VS ROI). Results shown are uncorrected at $p < .05$. Significant clusters are listed in Table 4.

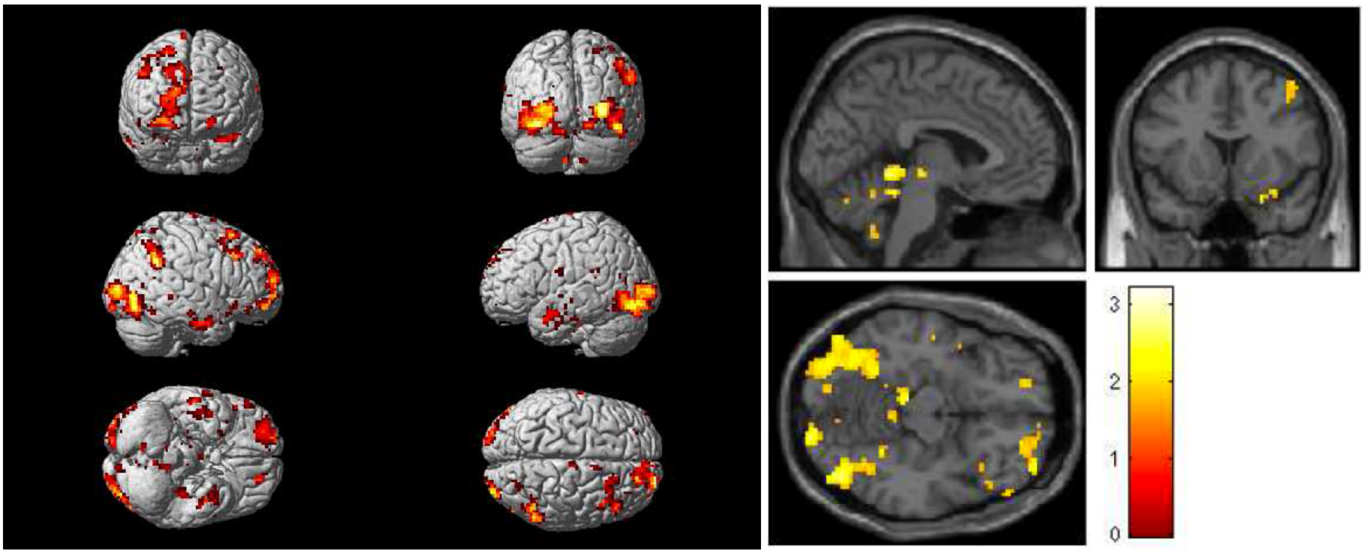

Supplementary Figure 2. Whole-brain functional activation for the emotion contrast (happy > neutral faces). The left panel shows activations in cortical areas, the right panel shows slices at $x = -4$, $y = 15$, $z = -13$ (the center of the VS ROI). Results shown are uncorrected at $p < .05$. The figure highlights activation mainly in right and left inferior occipital regions and the fusiform gyrus as well as right middle and superior frontal regions. None of the peaks was significant at cluster-level.

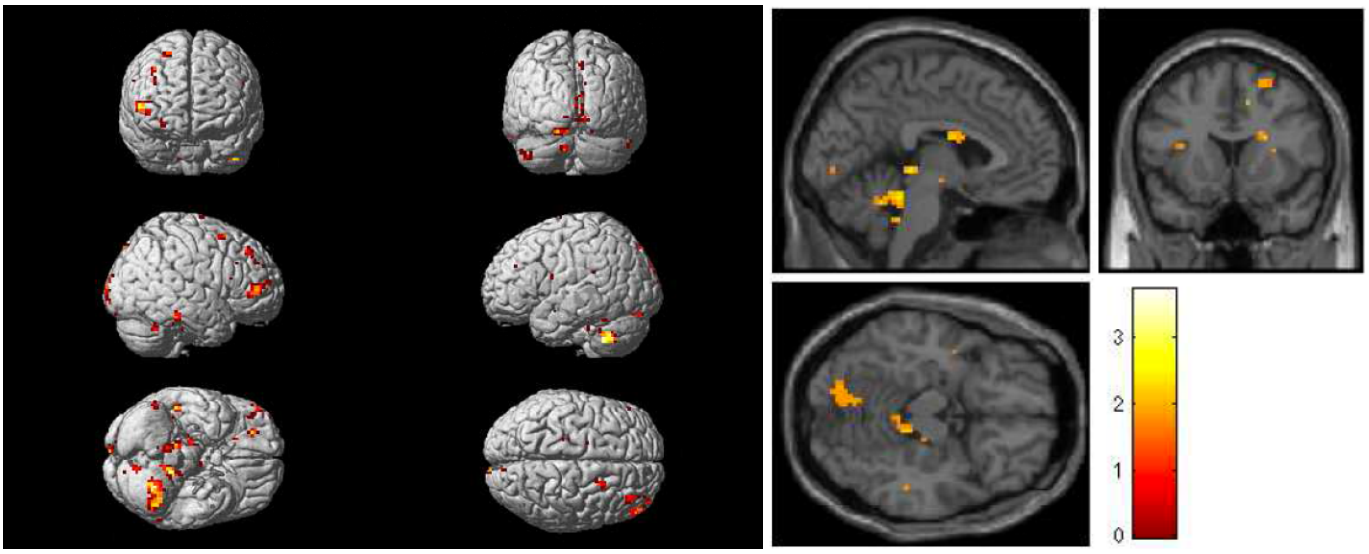

Supplementary Figure 3. Whole-brain functional activation for the interaction between inhibition and emotion. The left panel shows activations in cortical areas, the right panel shows slices at $x = -4$, $y = 15$, $z = -13$ (the center of the VS ROI). Results shown are uncorrected at $p < .05$. The figure highlights activation mainly in the right and left cerebellum, insula, caudate and putamen as well as the right parahippocampal gyrus. None of the peaks was significant at cluster-level.

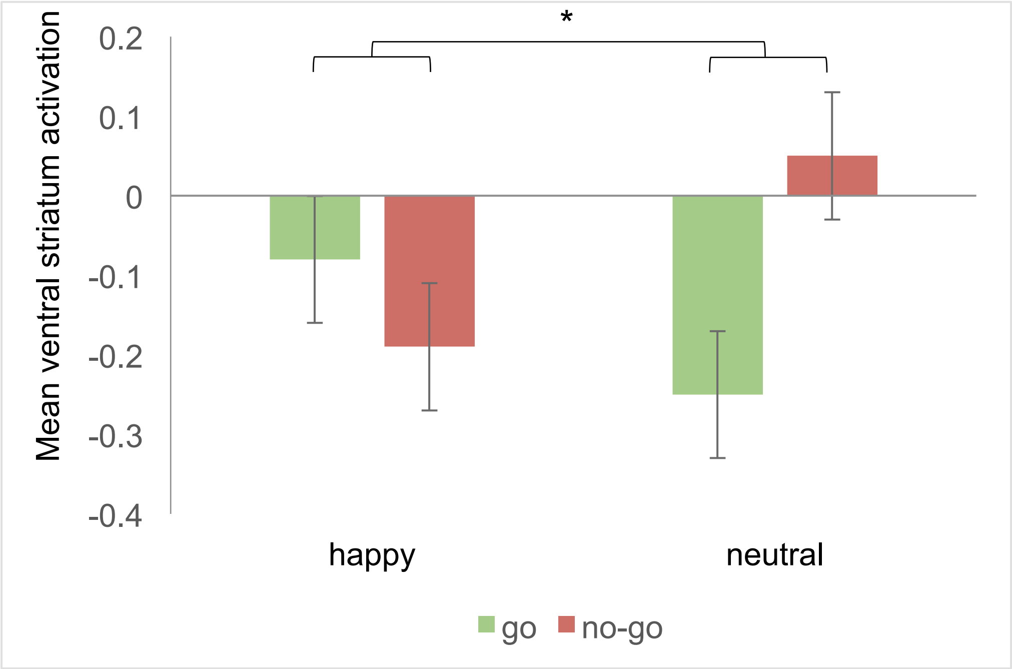

Supplementary Figure 4. Ventral striatum activation during the emotional go-no-go task. Mean predicted, MarsBaR-extracted, signal with standard error bars are shown for responding (go) and withholding responses (no-go) to happy and neutral faces (* $p < .05$).

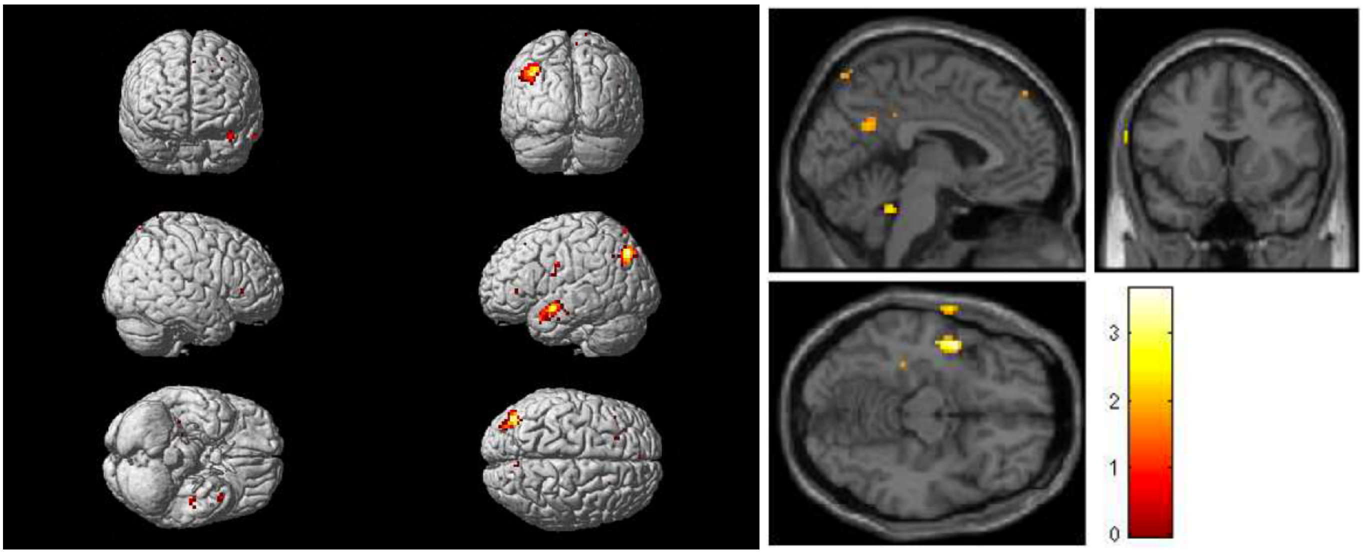

Supplementary Figure 5. Whole-brain connectivity for the inhibition contrast (no-go > go trials). The left panel shows activations in cortical areas, the right panel shows slices at $x = -4$, $y = 15$, $z = -13$ (the center of the VS ROI). Results shown are uncorrected at $p < .05$. The figure shows activation mainly in left middle temporal and left middle occipital regions as well as the posterior cingulate gyrus. None of the peaks was significant at cluster-level.
